# Fertility Stress, Psychological Resilience, and Depressive Symptoms in Women With Polycystic Ovary Syndrome

**DOI:** 10.7759/cureus.70352

**Published:** 2024-09-27

**Authors:** Zixuan Zhang, Meiju Liu, Fei Zhao, Hong Chen, Xinxia Chen

**Affiliations:** 1 School of Nursing and Rehabilitation, Shandong University, Jinan, CHN; 2 Department of Reproductive Medicine, Linyi People's Hospital, Linyi, CHN; 3 Department of Outpatient Clinic, Linyi People's Hospital, Linyi, CHN

**Keywords:** depressive symptoms, fertility stress, mediation model, pcos, psychological resilience

## Abstract

Background: Infertility is a common problem for patients with polycystic ovary syndrome (PCOS), which is closely related to stress and raises the risk of depression, while psychological resilience has been revealed to be protective for mental health. However, the associations of fertility stress, psychological resilience, and depressive symptoms in individuals with PCOS are not thoroughly explored. Our study aims to examine the associations of fertility stress and psychological resilience with depressive symptoms among patients with PCOS, as well as the mediating role of psychological resilience.

Methods: A cross-sectional study was conducted in a reproductive clinic. The participants completed structured questionnaires on fertility stress (Fertility Problem Inventory (FPI)), psychological resilience (10-Item Connor-Davidson Resilience Scale (CD-RISC 10)), and depressive symptoms (Beck Depression Inventory-II (BDI-II)). Hierarchical regression was utilized to explore the relationship between fertility stress, psychological resilience, and depressive symptoms in PCOS patients after controlling for covariates. Psychological resilience was included in the final layer of the regression model to determine its potential mediating roles by comparing changes in the effect sizes between models. The percentage of mediating effect was then determined using structural equation modeling (SEM).

Results: A total of 192 women with PCOS were included. Of them, 50 (26%) presented depressive symptoms, 18% of which were severe. Hierarchical regression showed that after adjusting for sociodemographic and health-related characteristics, both fertility stress (β=0.361; p<0.001) and resilience (β=-0.453; p<0.001) were significantly correlated with depressive symptoms. SEM demonstrated that fertility stress was directly linked to depressive symptoms in women with PCOS (β=0.050; 95% CI (0.028, 0.084); p<0.001). Psychological resilience mediated 21.9% of the relationship between fertility stress and depressive symptoms (β=0.014; 95% CI (0.005, 0.034); p=0.001).

Conclusion: This study demonstrated that among women with PCOS, fertility stress was, directly and indirectly through psychological resilience, associated with depressive symptoms. However, this study was cross-sectional, and the participants were recruited from a single clinical hospital. If replicated in longitudinal studies, the findings provide fertility stress and psychological resilience as potential intervention targets for better mental health in this population.

## Introduction

Polycystic ovary syndrome (PCOS) is a complex endocrine condition with a high incidence, affecting 4-21% of women at reproductive age [1]. There is growing evidence that women with PCOS have a higher depression risk, with four times more likely to have depressive symptoms than those without the condition [2]. Depression negatively impacts fertility [3], which would be particularly challenging for women with PCOS who already have difficulty getting pregnant due to irregular menstruation. However, factors that contribute to depressive symptoms in PCOS are largely unknown. Prior research has investigated the significance of pathological features like obesity, hirsutism, acne, and menstrual disorders [4]. Nevertheless, less attention has been paid to psychological contributors. Moreover, PCOS phenotypes differ significantly between ethnicities, which may result in different degrees of distress as well as influencing factors for PCOS patients. Most previous studies were performed in Hispanic women, but it remains unclear whether these pathological factors are also implicated in depressive symptoms among Asian women with PCOS. Therefore, it is essential to explore the influence of psychological contributors on depressive symptoms in patients with PCOS.

Fertility-related stress refers to the stress that patients experience from social, relationship, and family aspects due to poor fertility status or fertility problems, which includes sexual concerns, relationship concerns, social concerns, the need for parenthood, and the rejection of a child-free lifestyle [5]. Because of anovulation and irregular menstruation, women with PCOS frequently face difficulties in getting pregnant and therefore suffer from significant fertility-related stresses [6]. Several studies have shown that stress has a significant role in the low quality of life that infertile women experience [7]. However, the relationship between specific fertility-related stress and depressive symptoms in individuals with PCOS has not been studied. 

Furthermore, current research mainly focuses on risk factors for depressive symptoms in patients with PCOS, and less is known about the potential protective factors. Although exposed to highly stressful events, not all patients with PCOS will develop depressive symptoms, which may be related to protective factors in the face of stress. Psychological resilience is the capacity to reduce poor response to trying circumstances and a dynamic process of personal growth and success in the face of stress and hardship [8]. Previous research has revealed that those with high psychological resilience experience stressful situations less depressed [9]. Nevertheless, the association of psychological resilience with depressive symptoms among patients with PCOS remains to be investigated.

In addition, resilience has been proposed as the reserve of physiological systems that guard against and counteract the effect of adverse events, which can change and scale across an individual's lifespan [10]. In infertile women, high fertility stress was associated with low resilience and increased risk of poor mental health [11], suggesting that resilience reserve may be attenuated by stress. Less psychological resilience has been noted in individuals with PCOS in comparison to healthy controls [12]. The role of resilience in modulating the relationship between fertility stress and depressive symptoms, however, has not been studied.

Therefore, this study aims to investigate the associations of fertility stress and psychological resilience with depressive symptoms and the mediating role of psychological resilience in the relationship between fertility stress and depressive symptoms among patients with PCOS. Three hypotheses are proposed: In H1, PCOS patients' fertility stress is positively associated with depressive symptoms. In H2, PCOS patients' psychological resilience is negatively associated with depressive symptoms. In H3, PCOS patients' psychological resilience mediates the relationship between fertility stress and depressive symptoms.

## Materials and methods

Study design and population definitions

We carried out a cross-sectional research of patients with PCOS (ages 18-40 years old) attending reproductive centers. According to the Rotterdam Criteria [13], PCOS was diagnosed by two out of three criteria: hyperandrogenism, oligo-/anovulation, and polycystic ovaries under ultrasound (a follicle number per ovary ≥12 and/or ovarian volume >10 ml). Excluded were those with severely diagnosed mental illness or serious other systemic complications or who refused to complete the questionnaire. This study was approved by the School of Nursing and Rehabilitation Ethics Review Committee at Shandong University (approval number: 2021-R-153). It was guaranteed that personal information would remain private.

Sample size estimation

Sample sizes in this study were estimated based on the two research objectives. For the first objective of determining the association between fertility stress, psychological resilience, and depressive symptoms, the required sample size was calculated based on multifactorial analysis, which requires at least 10 times the pre-analyzed variable [14]. A total of nine predictive factors were considered in this study, with a sample size of at least 90, and after taking into account for 15% invalid rate, at least 106 cases should be included. For the second objective of testing the mediation effects of psychological resilience, structural equation modeling (SEM) was used for which a sample size of 200 was recommended [15]. Accounting for 15% invalid responses, a sample size of 235 was estimated. To sum up, at least 235 samples should be included.

Sampling method and participant recruitment

Trained research assistants stayed at the reproductive center to invite eligible PCOS patients to participate in this study. To minimize sampling bias, all patients with confirmed PCOS diagnosis were included in the study if they met the inclusion criteria, regardless of region, ethnicity, or age. The inclusion of study participants strictly followed the principle of voluntary, informed, and beneficial. When explaining the study to eligible women, it was emphasized that their decision to participate or not would not affect subsequent treatment and if they participated, all information would be kept strictly confidential. All subjects signed informed consent before joining the study. After that, a series of paper-pencil questionnaires were administered, which were finished face-to-face and retrieved upon completion. For women with severe depressive symptoms on the Beck Depression Inventory-II (BDI-II) scale, timely referral services are provided. Ultimately, a total of 242 surveys were collected, of which 50 were excluded due to missing information or untrue responses (consistent answers across the whole questionnaire or regularity between answers).

Measure tools and data collection

Women with PCOS were given a series of questionnaires examining fertility stress, depressive symptoms, and psychological resilience, using validated tools. Moreover, information on socio-demographics (age, education, and family economic status) and health state (BMI, hirsutism, and acne) were collected based on self-designed questions.

The Fertility Problem Inventory (FPl) is a 46-item questionnaire which includes five subscales: social concern, relationship concern, the need for parenthood, sexual concern, and rejection of a child-free lifestyle. A higher score indicates more severe fertility stress. The FPl has been widely used around the world to assess the impact of infertility. It was translated and adapted in a Chinese context by Peng et al. in the 2011 year [5]. The Chinese version showed moderate to high internal consistency in all items and satisfactory validity and has been used in infertile and PCOS populations. In our sample, Cronbach's alpha was 0.751.

The BDI-II is a 21-item questionnaire. Scores on 21 items added are the total score, which is divided into four levels: 0-13=no depression, 14-19=mild depression, 20-28=moderate depression, and 29-63=severe depression. It was previously shown that the Chinese version of BDI-II was determined to have high internal consistency and test-retest reliability, similar to the English version, and has been used in women with PCOS with a Cronbach's alpha of 0.915 [16,17]. In the current study, the Cronbach's alpha value of BDI-II was 0.902.

The 10-Item Connor-Davidson Resilience Scale (CD-RISC 10) is a 10-item questionnaire, with a higher score indicating higher psychological resilience. The Mandarin version of the CD-RISC 10 has been reported to have adequate reliability and validity, which has been used in infertile women with good internal consistency [18]. In the present study, the Cronbach's alpha value of CD-RISC 10 was 0.940.

Variables that may affect the exposure and the outcome, directly or indirectly, were identified from previous literature [19-22]. The minimal set of covariates controlled in this study was determined based on previous literature and expert experience. Age and BMI (kg/m^2^) were included as continuous variables; education (≤9, 10-12, 13-16, ≥17), income (<10000 y/m, 10000-30000 y/m, >30000 y/m), hirsutism (no, mild, moderate, severe), and acne (no, mild, moderate, severe) were included as categorical variables.

In this study, all research assistants received comprehensive training on the study protocol, data collection techniques, and ethical considerations before the formal survey. All women diagnosed with PCOS were invited to participate in the study to encompass all phenotypes, and a series of paper-pencil questionnaires were administered instead of online surveys, which were retrieved face-to-face.

Statistical analysis

IBM SPSS Statistics for Windows, Version 25.0 (Released 2017; IBM Corp., Armonk, New York, United States) was used to perform statistical analysis. The continuous variables were presented as mean and standard deviation. The categorical variables were presented as frequency and percentages. The associations of fertility stress, psychological resilience, and depressive symptoms were examined using Pearson's correlation analysis. A hierarchical regression model was created with depressed symptoms as the dependent variable and general information, fertility stress, and psychological resilience as the independent variables, to investigate the impact of fertility stress and psychological resilience on depressive symptoms in PCOS. Model 1 was adjusted for age, educational background, family economic status, and three body image-related variables: BMI, hirsutism, and acne. Model 2 was additionally modified for fertility stress. Finally, psychological resilience was added to Model 3. When psychological resilience was finally included in the model, statistically significant changes were revealed in effect sizes, indicating a potential mediating role of psychological resilience between fertility stress and depressive symptoms.

Finally, the mediating role of psychological resilience on the association between fertility stress and depressed symptoms was tested using SEM by Amos Graphics 24. Based on statistical theory and research consensus [23-25], the acceptable model fit in the SEM analysis is the following: 1< CMIN/df <3, RMSEA <0.08, CFI >0.90, TLI >0.90, and IFI >0.90. Specifically, CMIN is the chi-squared value, which reflects the extent to which the model differs from the data, while df is the degree of freedom, which indicates the number of parameters. A larger value of CMIN/df indicates a worse fit to the data. It is generally recognized that a model with a CMIN/df <3 is acceptable, <1 indicates overfitting, and >5 indicates poorly fitted. RMSEA is a badness-of-fit index (larger values indicate worse fit) ranging from 0.0 to 1.0. It has been suggested that RMSEA <0.08 is acceptable and <0.05 is better, while >0.1 indicates poor model fit. CFI, TLI, and IFI are goodness-of-fit indexes ranging from 0.0 to 1.0, and larger numbers are better. It is generally accepted that a model with CFI, TLI, and IFI >0.95 is good and >0.9 is acceptable.

## Results

Baseline description

The investigation comprised 192 patients with PCOS, with an average of 28.29±2.92 years old. Depressive symptoms (BDI-II >13) were presented in 26% of individuals with PCOS, with 18% classified as severe. Mean scores were 153.57±19.80 for fertility stress and 27.95±8.59 for psychological resilience. And 40.1% of the participants had a BMI within the normal range. More than two-thirds of the participants had no or mild acne or hirsutism scores (Table 1).

**Table 1:** Sample characteristics of women with PCOS (n=192) SD: standard deviation; BDI-II: Beck Depression Inventory-II; PCOS: polycystic ovary syndrome

Variable	N (%)	Mean (SD)
Depression (BDI-II >13)	50 (26)	-
Mild (14-19)	23 (46)	-
Moderate (20-28)	18 (36)	-
Severe (29-63)	9 (18)	-
Depressive symptoms (continuous)	-	9.42 (9.14)
Fertility stress (continuous)	-	153.57 (19.80)
Psychological resilience (continuous)	-	27.95 (8.59)
Age (years)	-	28.29 (2.92)
BMI (kg/m^2^)	-	25.40 (3.85)
Normal (18.5-23.9)	77 (40.1)	-
Overweight (24-27.9)	61 (31.8)	-
Obesity (≥28)	49 (25.5)	-
Education (years)		
≤9	68 (35.4)	-
10-12	58 (30.2)	-
13-16	65 (33.9)	-
≥17	1 (0.5)	-
Income (y/m)		
<10000	8 (4.2)	-
10000-30000	153 (79.7)	-
>30000	31 (16.2)	-
Hirsutism		
No	53 (27.6)	-
Mild	72 (37.5)	-
Moderate	60 (31.3)	-
Severe	7 (3.7)	-
Acne		
No	131 (68.2)	-
Mild	48 (25)	-
Moderate	12 (6.3)	-
Severe	1 (0.5)	-

Association of fertility stress, psychological resilience, and depressive symptoms

Pearson's correlation analysis showed fertility stress and psychological resilience were substantially correlated with depressed symptoms (r=0.38, -0.50; p<0.01) (Table 2). Three dimensions of fertility stress including relationship concern, sexual concern, and rejection of a child-free lifestyle (r=-0.27, -0.31, -0.29, respectively; p<0.01, respectively) showed a significantly negative association with psychological resilience, while every dimension of fertility stress was significantly positively correlated with depressive symptoms in patients with PCOS (Table 2).

**Table 2:** Associations of fertility stress, psychological resilience, and depressive symptoms SD: standard deviation; *: p<0.05 (significant); **: p<0.01 (significant)

	M (SD)	1	2	3	4	5	6	7
1. Psychological resilience	27.95 (8.59)	-	-	-	-	-	-	-
2. Fertility stress	153.57 (19.80)	-0.13	-	-	-	-	-	-
3. Social concern	33.11 (4.54)	-0.13	0.77**	-	-	-	-	-
4. Relationship concern	32.48 (5.70)	-0.27**	0.73**	0.52**	-	-	-	-
5. The need for parenthood	39.57 (7.39)	-0.13	0.72**	0.53**	0.53**	-	-	-
6. Sexual concern	24.86 (5.70)	-0.31**	0.75**	0.60**	0.53**	0.53**	-	-
7. Rejection of a child-free lifestyle	24.86 (5.70)	-0.29**	0.33**	0.06	-0.03	-0.15*	-0.06	-
8. Depression	9.42 (9.14)	-0.50**	0.38**	0.26**	0.44**	0.39**	0.48**	-0.22**

After controlling for covariates like BMI, hirsutism, and acne as well as other potential confounders, the hierarchical regression model indicated that fertility stress (β=0.361; p<0.001) positively whereas psychological resilience (β=-0.453; p<0.001) negatively predicted depressive symptoms in patients with PCOS. Notably, when psychological resilience was further added to the model, the regression coefficient of fertility stress on PCOS depressive symptoms decreased but remained significant (β=0.299; p<0.001), indicating that psychological resilience may play a mediating role between fertility stress and depressive symptoms (Table 3).

**Table 3:** Hierarchical multiple regression between fertility stress, psychological resilience, and depressive symptoms P<0.05 (significant)

	B	SE	β	T	P	R^2^	Adjusted R^2^	R^2^ change	F change	Significant F change
Model 1	-	-	-	-	-	0.038	0.006	0.038	1.202	0.307
(Constant)	11.590	8.795	-	1.318	0.189	-	-	-	-	-
Age	-0.096	0.231	-0.031	-0.415	0.679	-	-	-	-	-
Education	0.041	0.825	0.004	0.050	0.960	-	-	-	-	-
Income	-2.984	1.591	-0.142	-1.875	0.062	-	-	-	-	-
BMI	0.155	0.176	0.065	0.881	0.380	-	-	-	-	-
Hirsutism	0.939	0.796	0.087	1.180	0.240	-	-	-	-	-
Acne	0.621	1.114	0.042	0.557	0.578	-	-	-	-	-
Model 2	-	-	-	-	-	0.164	0.132	0.126	27.541	0.000
(Constant)	-14.251	9.584	-	-1.487	0.139	-	-	-	-	-
Age	-0.044	0.216	-0.014	-0.202	0.840	-	-	-	-	-
Education	-0.230	0.773	-0.021	-0.298	0.766	-	-	-	-	-
Income	-2.044	1.498	-0.097	-1.364	0.174	-	-	-	-	-
BMI	0.069	0.166	0.029	0.414	0.680	-	-	-	-	-
Hirsutism	0.627	0.746	0.058	0.841	0.402	-	-	-	-	-
Acne	0.753	1.042	0.051	0.723	0.471	-	-	-	-	-
Fertility stress	0.167	0.032	0.361	5.248	<0.001	-	-	-	-	-
Model 3	-	-	-	-	-	0.360	0.331	0.196	55.692	0.000
(Constant)	7.624	8.905	-	0.856	0.393	-	-	-	-	-
Age	-0.199	0.191	-0.064	-1.046	0.297	-	-	-	-	-
Education	0.428	0.684	0.039	0.626	0.532	-	-	-	-	-
Income	-2.343	1.315	-0.112	-1.782	0.076	-	-	-	-	-
BMI	0.069	0.145	0.029	0.473	0.637	-	-	-	-	-
Hirsutism	0.593	0.655	0.055	0.905	0.366	-	-	-	-	-
Acne	0.615	0.915	0.042	0.673	0.502	-	-	-	-	-
Fertility stress	0.138	0.028	0.299	4.901	<0.001	-	-	-	-	-
Psychological resilience	-0.482	0.065	-0.453	-7.463	<0.001	-	-	-	-	-

Mediation analysis

**Figure 1:**
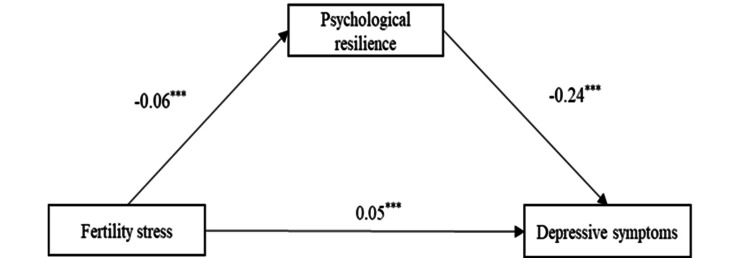
Structural equation modeling of the mediating role of psychological resilience in the relationship between fertility stress and depressive symptoms. Standardized path coefficients are shown on the unidirectional arrow paths ***: p<0.001 (significant)

We further explored how psychological resilience affected the relationship between fertility stress and depressed symptoms using SEM (Figure 1). As seen in the results, fertility stress was directly associated with depressive symptoms (β=0.050; 95% CI (0.028, 0.084); p<0.001; Table 4), and psychological resilience significantly mediates the relationship between fertility stress and depressed symptoms (β=0.014; 95% CI (0.005, 0.034); p=0.001; Table 4). The standardized total effect of psychological resilience on depressive symptoms is 0.064, and the standardized mediating effect of psychological resilience between fertility stress and depressed symptoms is (-0.06)×(-0.24)≈0.014. Thus, the ratio of mediating effect to total effect is 0.014/0.064≈21.9%.

**Table 4:** The mediating effect of psychological resilience on the association of fertility stress and depressive symptoms in women with PCOS P<0.05 (significant) PCOS: polycystic ovary syndrome

	Effect	95% Boot LLCI	95% Boot ULCI	P
Total	0.064	0.040	0.104	0.001
Direct effect	0.050	0.028	0.084	<0.001
Indirect effect	0.014	0.005	0.034	0.001

## Discussion

To our knowledge, this is the first study that looks at protective characteristics and how resilience regulates the relationship between fertility stress and depressed symptoms in PCOS. This study revealed that fertility stress positively while psychological resilience negatively predicted depressive symptoms in individuals with PCOS, which remained significant after accounting for BMI, hirsutism, and acne. Moreover, a portion of the relationship between reproductive stress and depressed symptoms was mediated by psychological resilience. The results indicated that boosting psychological resilience, in addition to decreasing fertility stress, might be helpful in reducing depressive symptoms in patients with PCOS, which is expected to provide a key intervention target for the advancement of mental health in PCOS.

The present findings are consistent with earlier literature, which indicates that women with PCOS were experiencing significant mental health issues. Specifically, nearly one-third of women with PCOS experience depressive symptoms, with more than half of them experiencing moderate to severe depression. A meta-analysis showed that women with PCOS in China reported having more depression than women in other countries [26]. This study, in conjunction with previous research, emphasizes the need to find effective methods for the significant mental health issues experienced by PCOS-afflicted women.

As expected, the current study found that fertility stress, all and in each dimension including social concern, relationship concern, the need for parenthood, sexual concern, and rejection of a child-free lifestyle, was strongly correlated with depressive symptoms in patients with PCOS. These findings supported earlier research that indicated stress was a risk factor for psychological issues in the general population [27]. An earlier study conducted on infertile women also showed that stress related to infertility was positively correlated with depression [28]. A study reported that stress played an important role in depressive symptoms among women with PCOS; however, it assessed stress in daily life rather than fertility-related stress [29]. We are aware of only one study that examined the impact of reproductive stress on women with PCOS and found that it was negatively connected with the quality of life related to PCOS symptoms [6]. Nevertheless, some important factors including the severity of acne and hirsutism were not accounted for. Our study confirmed stress as a key factor for depressive symptoms of women with PCOS after adjusting for previously overlooked factors and highlighted fertility stress in particular.

Furthermore, our results extended that fertility stress significantly predicted depressive symptoms even after adjusting for common body image concerns including BMI, acne, and hirsutism. In the study by Alur-Gupta et al. [30], body image concern was revealed to completely explain depressive symptoms in women with PCOS, which was different from the present study, indicating that stress is common among the PCOS population while the specific stress that contributes to adverse mental health might vary between ethnicities or countries. According to the findings by Sheikh et al. [31], Chinese women may have more fertility concerns, while White women and those born in the United Kingdom have higher rates of body image concerns and weight stigma.

One possible explanation is the influence of traditional cultural background and fertility philosophy. Chinese women place high importance on fertility because they view it as a crucial foundation for the transmission of the bloodline and the realization of the value of motherhood, and fertility-related problems may be an important source of psychological distress. Childlessness or reduced reproductive function results in a lack of parental role and low sexual function, which can prevent a family from functioning normally, escalating conflict within the couple and a crisis in the marriage [32]. Thus, fertility stress puts Chinese women with PCOS under pressure not only for social daily life but also for relationship concerns, sexual concerns, and role demands. Stress related to fertility may contribute more to psychological disorders in PCOS than the stress of daily life. 

Importantly, this study found psychological resilience as a protective factor in PCOS for the first time. This result is consistent with existing research in the general population. People with strong psychological resilience tend to have a more tenacious, self-improving, and optimistic attitude and are more likely to respond in a positive way to difficult conditions. A study based on a national population suggested that resilience was associated with mental health and resilience significantly predicted depression in young people [33]. Furthermore, it has been proved that enhanced resilience can buffer the impact of stress on the mental health of patients with endocrine and metabolic disorders, such as diabetes [34]. As a result of the pathophysiological characteristics of PCOS, including the hypothalamic-pituitary-adrenal (HPA) axis, immune system inflammation, and hyperactivity of the sympathetic nervous system (SNS), affected women may be more susceptible to stress. The current study proved that resilience could alleviate the negative effect of fertility stress on depressive symptoms.

Notably, resilience is a characteristic that can be developed and enhanced. Previous research indicated that resilience training, such as psychoeducation or didactic introductions, mindfulness techniques, and relaxation techniques, significantly increased resilience and decreased depressive symptoms in cancer patients [35]. Also, in endocrine or metabolic diseases such as diabetes, interventions aimed at improving psychological resilience have been found to have significant effects on mental concerns [36]. Given that resilience could mediate more than 20% of the effect of fertility stress on depressive symptoms, future studies are warranted to examine the effectiveness of resilience as an intervention target in women with PCOS.

In addition, this study did not find significant associations of hirsutism, acne, and BMI with depressive symptoms in individuals with PCOS, contrary to prior studies mainly performed in Hispanic women [4]. The reason for this may be related to PCOS phenotypes. The PCOS phenotype in Chinese women is characterized by a higher prevalence of menstrual irregularities and ovarian cysts, rather than hirsutism and elevated androgen levels [37]. Also, previous studies reporting significant relationships between BMI and depressive symptoms have mostly been performed in PCOS with high BMI levels or large proportions being obese [38], while in Asia PCOS patients have a relatively normal BMI and fewer are obese. Our opinion was supported by a study on the mental health of Chinese patients with PCOS, which revealed no correlation between mental health issues and any illness states, such as hirsutism, acne, or BMI. These results suggest that factors contributing to poor mental health among women with PCOS may differ between phenotypes or ethnicities.

Strengths and limitations

This study focuses on a specific population, looking at the mental health of women with PCOS and searching for key targets that can be improved. Stress is universal, while, unlike the previous focus on general stress, fertility-specific stress is a risk factor that women with PCOS should focus on additionally. More importantly, this study found that resilience plays an important role in preventing depressive symptoms and mediates the relationship between fertility stress and depressive symptoms, which has implications for policy and practice. Enhanced psychological resilience should be an important intervention point when designing prevention programs targeting depressive symptoms in women with PCOS. Health education programs targeting women's mental health should enhance the awareness of self-protective psychological factors. For instance, early implementation of mindfulness training and relaxation exercises has been found to be of significant importance in enhancing psychological resilience and decreasing the symptoms of depression.

There are still several limitations in the study that should be considered. First, cross-sectional studies fail to confirm causality; only the correlation between dependent and exposure factors can be identified. The possibility of a reverse relationship that depression increases fertility stress cannot be excluded, and there might be bidirectional correlations between them. Future studies are warranted to examine the longitudinal relationship between fertility stress, psychological resilience, and depressive symptoms among women with PCOS, so as to provide evidence for prevention and intervention. Second, the study was performed on women with PCOS receiving fertility treatment from a single clinical hospital and conducted in a Chinese cultural context which emphasizes children as essential for marriage and family, which may restrict the generalizability of our findings. Given that fertility issues are common in the world regardless of culture, further validation of the findings in multiple cultural contexts is necessary in the future. Third, although our initial sample met the requirements for statistical analysis, the final sample size was slightly lower than required due to the higher invalid questionnaire rate, and the robustness of this result warrants verification in the future. Fourth, although the analyses were adjusted for a wide range of covariates, there might be confounders that were not included. Finally, data collection was performed during the pandemic, which might affect the mental health of patients. Future studies are warranted to validate the association between fertility stress, psychological resilience, and depressive symptoms among women with PCOS.

## Conclusions

The study demonstrated that among women with PCOS, fertility stress was, directly and indirectly through psychological resilience, associated with depressive symptoms. If replicated in longitudinal studies, the findings provide fertility stress and psychological resilience as potential intervention targets for better mental health in this population.

Although the study has limitations, such as establishing causality and challenges in generalizability, it offers valuable insights into PCOS patients' mental health. Future research should focus on longitudinal studies and in different populations, providing a deeper understanding of mental health in women with PCOS.
